# Dr. Searle's 'Why and Wherefore'

**Published:** 1847-04

**Authors:** 


Art. II.—*THE WHY AND THE WHEREFORE; or, the Philosophy of Life, Health, and Disease. New and Original Views explanatory of their Natural Causes and Connexion; and of the Treatment of Disease upon a few General Principles, based upon the Laws of Nature and Common Sense; with Rules for the Preservation of Health and Renovation of the System. The Fruit of Thirty Years' Observation and Experience.* By Charles Searle, m.d., m.r.c.s.e., and late of the E. I. C. Madras Establishment.—*London,* 1846. 8vo, pp. 266.

Dr. Searle dates his book from Bath; we infer, therefore, that he is practising as a physician there. He proposes to present the reader "with a complete system of the science and practice of medicine, and of the philosophy of life and health." His reader is not, however, expected to be the professional and educated man, and therefore a competent judge of the author's merits as a writer and as a practitioner, but rather the uneducated (we mean technically uneducated) layman. This is amply shown by the following long-winded paragraph:

"Being of opinion that the principles of the subject are within the comprehension of every intelligent person, and that a distinct knowledge of the principles of any subject is essential to its successful practice; and seeing the lamentable ignorance that exists in these matters, and the charlatanism which prevails, I have been induced to address myself to the public rather than to the profession, not from any disrespect to its members—far otherwise, for a more enlightened and liberal-spirited class of men nowhere exists—but with the view of laying open to the public the delusions of incompetent pretenders, and of imparting that amount of knowledge which every individual ought to possess, on a subject of such pre-eminently personal importance, and especially so as regards the causes and prevention of disease, and of those calamities we see daily recorded in the public journals, such as persons falling down dead in the streets." (Preface, p. v.

We think this a fundamental and fatal mistake in the plan of Dr. Searle's work, for mistake we believe it to be, and not a premeditated attempt at quackery. This is much to be regretted ; for if Dr. Searle had directed the energy and industry which he evidently possesses to a more judicious plan, he might have done good service to the public and the profession ; but as the matter stands, his book is too abstruse for the general reader, and too theoretical and generalizing for the professional reader; so his labours go for nothing. Dr. Searle might have written in elegant language on dietetics ; he has written *de omnibus rebus et quibusdam aliis.* He might have made the established principles of pathology popular and intelligible; he has attempted to make popular some theoretical notions of his own. He might have laid down a safe system of elementary therapeutics; he has, we fear, made public a somewhat dangerous system—and on this point we need only refer his recommendation of opium as a remedy in mental anxiety. He might at least have written scholarly; he has written with "mugginess," to use a word we see in print for the first time in his book.

An example of "muggish" writing, addressed, be it observed ; to "the public," for the noble purpose set forth in the foregoing quotation, is subjoined.

"153. Hysterical breathing or other spasmodic affection of the muscles of the tongue, the voice, or those muscles associated in swallowing, (circumstances concomitant with the mental excitement, and connected with the irritation in the brain involving that portion of it from which the nerves of these organs arise—the medulla oblongata and summit of the spinal marrow—inducing the patient to protrude his tongue and to utter discordant sounds, occasioning difficulty of swallowing or breathing,) if not to be relieved by a full dose of opium, which should be administered if necessary, exhibit the advanced condition of inflammation, when cupping or leeching the back of the head to a small extent daily, and a blister kept open between the shoulders or behind the ears, are the proper remedies, following them up by the insertion of a seton at the summit of the neck." (p. 114.)

Very explicit and useful information for "the public" here, and no less elegantly conveyed to the same "public"! But what do our readers think is the mode recommended to the decrepid "public" for the "renovation of the system"? Why just repeated bloodletting!

366. "*Renovating influence of bloodletting.* If what I have said is correct, we have, in the renewal of the stream by bloodletting, which not only removes the bad, but facilitates the entrance of the good and fresh materials, (absorption both of air and of nutriment being increased *in proportion as the vessels are emptied,*) [!!] a most valuable agent of renovation of the system, and one which, if judiciously employed, bids fair, I am of opinion, to relieve, if not to restore to perfect health, a large proportion of the decrepid and incurable which are everywhere to be found, but it will be argued, &c." (p. 257.)

Now we feel certain that Dr. Searle would himself condemn such doctrines as this if propounded by another writer; or if old age and decrepitude should overtake him, it is not probable that he would submit to the treatment which he advises for others, and be daily bled.

It is always to us a painful duty to write in disparagement of any work which we believe to be the production of an honest, and well-meaning, and respectable practitioner, as we believe Dr. Searle to be, and we cannot but express our regret that he has published his views in their present form.

